# A Longitudinal Study of 25-Hydroxy Vitamin D and Parathyroid Hormone Status throughout Pregnancy and Exclusive Lactation in New Zealand Mothers and Their Infants at 45° S

**DOI:** 10.3390/nu10010086

**Published:** 2018-01-13

**Authors:** Benjamin J. Wheeler, Barry J. Taylor, Michel de Lange, Michelle J. Harper, Shirley Jones, Adel Mekhail, Lisa A. Houghton

**Affiliations:** 1 Department of Women’s and Children’s Health, Dunedin School of Medicine, University of Otago, Dunedin 9054, New Zealand; barry.taylor@otago.ac.nz (B.J.T.); shirley.jones@otago.ac.nz (S.J.); adel.mekhail@southerndhb.govt.nz (A.M.); 2 Paediatric Endocrinology, Southern District Health Board, Dunedin 9010, New Zealand; 3 Department Preventative and Social Medicine, University of Otago, Dunedin 9054, New Zealand; michel.delange@otago.ac.nz; 4 Department of Human Nutrition, University of Otago, Dunedin 9054, New Zealand; michelle.harper@otago.ac.nz (M.J.H.); lisa.houghton@otago.ac.nz (L.A.H.)

**Keywords:** vitamin D, parathyroid hormone, pregnancy, breastfeeding, lactation, infant

## Abstract

Vitamin D status and associated metabolism during pregnancy and lactation have been assessed in only a limited number of longitudinal studies, all from the northern hemisphere, with no infant data concurrently reported. Therefore, we aimed to describe longitudinal maternal and infant 25-hydroxy vitamin D (25OHD) and parathyroid hormone (PTH) status during pregnancy and up to 5 months postnatal age, in New Zealand women and their infants living at 45° S latitude. Between September 2011 and June 2013, 126 pregnant women intending to exclusively breastfeed for at least 20 weeks were recruited. Longitudinal data were collected at three time-points spanning pregnancy, and following birth and at 20 weeks postpartum. Vitamin D deficiency (25OHD < 50 nmol/L) was common, found at one or more time-points in 65% and 76% of mothers and their infants, respectively. Mean cord 25OHD was 41 nmol/L, and three infants exhibited secondary hyperparathyroidism by postnatal week 20. Maternal late pregnancy 25OHD (gestation 32–38 weeks) was closely correlated with infant cord 25OHD, *r*^2^ = 0.87 (95% CI (Confidence interval) 0.8–0.91), while no correlation was seen between early pregnancy (<20 weeks gestation) maternal and cord 25OHD, *r*^2^ = 0.06 (95% CI −0.16–0.28). Among other variables, pregnancy 25OHD status, and therefore infant status at birth, were influenced by season of conception. In conclusion, vitamin D deficiency in women and their infants is very common during pregnancy and lactation in New Zealand at 45° S. These data raise questions regarding the applicability of current pregnancy and lactation policy at this latitude, particularly recommendations relating to first trimester maternal vitamin D screening and targeted supplementation for those “at risk”.

## 1. Introduction

Pregnancy is a unique and demanding life stage in terms of vitamin D and calcium metabolism, due to the increased need for fetal development of mineralised structures, while maintaining optimal maternal status. This is particularly the case in the third trimester of pregnancy when peak fetal bone mineral accrual occurs [1,2]. 25-Hydroxyvitamin D (25OHD) readily crosses the placenta. As a result, 25OHD levels of mother-infant cord blood are positively correlated. Maternal vitamin D status during pregnancy plays a key role in establishing the size of their neonate reserves at birth [3,4,5], with supplementation studies during pregnancy demonstrating significantly improved infant status at birth and beyond [3,6,7].

The potential adverse consequences to the neonate of maternal vitamin D deficiency have been demonstrated, with both maternal vitamin D deficiency and lack of prenatal supplementation associated with an increased risk of infancy and childhood rickets [8,9,10,11]. In addition, other aspects of foetal and child bone health may also be impacted by maternal vitamin D status, including foetal growth [4], foetal bone accrual and subsequent bone size [12,13], and dental health including enamel hypoplasia [14] and dental caries [15]. These impacts of low maternal vitamin D status on foetal bone have been reported as early as 19 weeks gestation using high resolution 3D ultrasound showing a poorer fetal fermoral development [16], as well as longer-term follow up studies demonstrating lower peak offspring bone mass at 20 years of age [17].

Maternal vitamin D status during pregnancy is influenced by a range of factors, including season, skin colour, supplementation, latitude, and potential pregnancy specific variations in metabolism [4,18,19]. To better understand the magnitude of these factors, maternal vitamin D status and associated metabolism have been assessed in a number of longitudinal studies [19,20,21,22,23,24,25]. Nonetheless, all of the studies to date have been conducted in the northern hemisphere (spanning 39°–63.8° N) where supplement use is common. Many of these studies were also limited by unbalanced seasonal sampling or lack of seasonal data. In addition, post-pregnancy maternal follow-up during lactation was variable and the respective infant data was not reported.

A better understanding of vitamin D status and metabolism during pregnancy and lactation is needed, particularly in regions of the world where mothers are at risk of suboptimal vitamin D status. New Zealand is one such country due to its southern latitude (spanning 35–47° S), and negligible vitamin D food fortification. Pregnancy and infant vitamin D deficiency (25OHD < 50 nmol/L) in New Zealand also appear common with high rates reported across diverse ethnicities and latitudes [18,26,27,28]. In addition, current public health policy is to only consider vitamin D supplementation to women during pregnancy and to breastfed infants who are ‘‘at risk” i.e., having 1 or more of the following: naturally dark skin; complete sun avoidance; a sibling with rickets; have liver or kidney disease, or on certain medications that affect vitamin D levels; and infants who are breastfed over winter [29].

Therefore, we aimed to describe longitudinal 25OHD and parathyroid hormone (PTH) status throughout pregnancy as well as 20 weeks postnatally in a sample of New Zealand women and their infants living at a latitude of 45° S.

## 2. Methods

### 2.1. Study Population and Design

This longitudinal descriptive study includes 126 pregnant women intending to exclusively breastfeed for at least 20 weeks, and their infants. Blood samples obtained antenatally during routine care were collapsed into the following three categories: (1) <20 weeks gestation “first trimester” screening bloods (with specific sample timing determined by when pregnancy was confirmed and antenatal care commenced); (2) 28-week maternal gestational diabetes screening (range 20–31 weeks); and (3) 36-week routine maternal iron status determination (range 32–38 weeks). There were two additional postnatal time points measured in both mothers and infants, at 4 and 20 weeks for mothers, and cord blood (at delivery) and 20 weeks for infants. Overall, 40 women provided all five maternal observations, 40 provided four observations, 24 provided two observations, and 22 women only one observation, thereby providing 431 maternal observations for analysis in total.

This sample population of women and their infants was drawn from those recruited and screened as part of a previously published randomised controlled trial (RCT). Detailed methods for this study have been published elsewhere [26]. In brief, healthy pregnant women planning to exclusively breastfeed for at least five months following delivery were recruited from September 2011 to June 2013 through the Queen Mary Maternity Centre (QMMC), Dunedin Hospital, Dunedin, New Zealand (45° S latitude). QMMC provides publicly funded, tertiary level maternity and newborn care to the Otago/Southland region of New Zealand (population ~320,000), and is the only birthing unit in Dunedin, with approximately 97% of all city births (the remaining 3% being home births). Exclusion criteria included: (1) delivery prior to 37 weeks gestation; (2) intent to use postnatal vitamin D supplements (mother or infant); (3) a history of disorders known to affect calcium and/or vitamin D metabolism, including abnormal calcium levels/urine Ca/Cr ratio at study baseline; and (4) planned travel outside of New Zealand over the study period.

The study consisted of a 16 week randomised, double-blind, placebo-controlled trial of 90 mother and infant pairs, beginning at 4 weeks postpartum. Following delivery, lactation support was provided, along with breast pumps as needed. At 4 weeks postpartum, mothers were randomised in blocks of 15 to one of three treatment arms: placebo, 50,000 IU, or 100,000 IU cholecalciferol to be administered every month until 16 weeks postpartum (inclusive). Women were enrolled from 20 weeks gestation until delivery, and antenatal data were regularly collected for subsequent analysis for this longitudinal study. Postnatal week 20 data for both mothers and infants for those in the two intervention arms of this study have been excluded for the purposes of this longitudinal study due to the potential impact of this intervention on their “free-living” vitamin D status.

**Figure 1:**
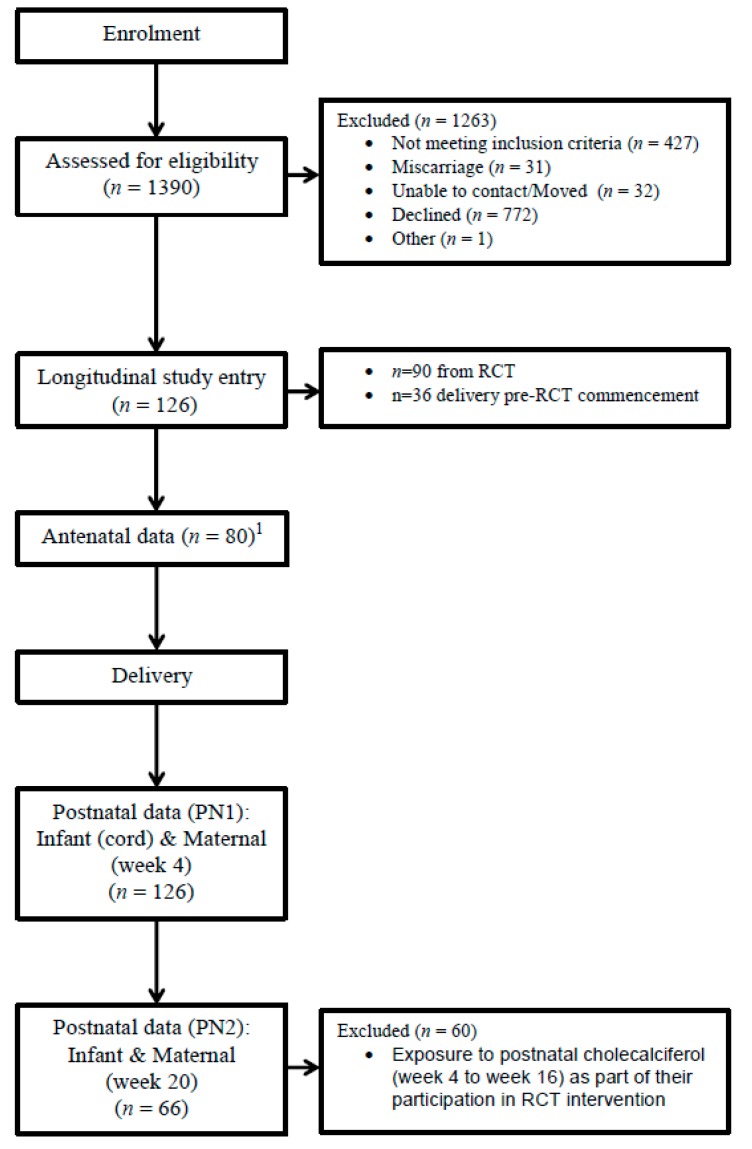
Diagram depicting longitudinal study participant flow.

In addition, in the present study, an additional 36 women who were recruited prior to RCT commencement (due to initial delays with the availability of independently verified placebo and intervention vitamin supplement) from September 2011 to June 2012, were also included. These women were enrolled from the same population, using identical inclusion/exclusion criteria (Figure 1).

Socio-demographic data were obtained from interviews at antenatal study enrolment (maternal only), and at 4 and 20 weeks postpartum (both mother and baby). Information was obtained on maternal age, education, ethnicity (in 7 instances dual ethnicities were given, in these cases for the purposes of description in this study, these were allocated to a single ethnic group based order of priority: Māori, Pacific, Asian and European/Other), smoking status (yes/no—pre-pregnancy and pregnancy), vitamin and mineral supplement use (recall of number of days/week taken during each month of pregnancy), breastfeeding and use of infant formula (frequency and duration), sun exposure (including degree of veiling/covering behaviour, and hours of exposure previous month), and sunscreen use (frequency). Maternal height and weight measurements were taken at enrolment and at 4 weeks post-partum using standardised techniques, and body mass index (BMI) was subsequently calculated. Infant length and weight measurements were taken at birth, 4, and 20 weeks of age. Lastly, maternal and infant skin reflectance was measured at each visit by spectrophotometer (CM2600d, Minolta Co. Ltd., Osaka, Japan) and converted to a final skin colour assessment by calculating individual typology angles (ITA) using the following formula: ITA = (ArcTangent ((*L* − 50)/*b*)) − 180/π. Skin colour was then classified using the ITA into the following groups: very light > 55 > light > 41 intermediate > 28 > dark > −10 > very dark. Measurement sites included the medial aspect of the upper arm (natural skin colour) and dorsal aspect of the forearm (sun exposed surface).

### 2.2. Dietary Vitamin D intake

From antenatal enrolment to week 20 postpartum, mothers recorded use of dietary supplements (if any), as well as the brand of infant formula used and daily volume consumed (if any) for the infant. These data were reviewed by study personnel at each study visit. Maternal supplemental vitamin D intake was then determined by estimating mean supplement intake (IU/day) over the duration of pregnancy (there was no postnatal use once the RCT commenced). No infant vitamin supplements were used. However, the amount of dietary vitamin D intake consumed by the infants was estimated from the mean daily intake of infant formula (mL/day) multiplied by the vitamin D content (IU/100 g) over the 20-week study period.

### 2.3. Blood Sampling and Laboratory Analysis

Non-fasting venous maternal, cord, and infant blood samples were collected, and stored at −80°C until study completion. Serum samples for 25OHD and PTH from each participant were analysed in the same run to avoid inter-assay variation. Serum levels of 25OHD were measured by isotope-dilution liquid chromatography tandem mass spectrometry [30], using an API 3200 instrument (Applied Biosystems) connected to a Dionex Ultimate 3000 HPLC system. The limit of quantification for the assay was <5 nmol/L for both metabolites. To assess accuracy and inter-assay variability, external quality control serum material (UTAK Laboratories) containing low and medium levels of both metabolites were analysed with every run. Measurements fell within the expected range with mean ± SD values of 29.0 ± 1.2 nmol/L and 67.3 ± 2.1 nmol/L for 25(OH)D3 (UTAK verified values 25.0, 69.9 nmol/L), and 26.2 ± 1.4 nmol/L and 67.0 ± 2.9 nmol/L for 25(OH)D2 (UTAK verified values 29.1, 67.9 nmol/L). Internal quality control pooled serum samples were also analysed, the inter-assay CV for 25(OH)D3 being 3.7% at 56.9 nmol/L, 25(OH)D2 was below the limit of quantification.

PTH was measured using an automated electrochemiluminescence immunoassay (Elecsys^®^2010, Roche Diagnostics, Mannheim, Germany). The PTH assay showed a detection sensitivity of 0.1 pmol/L. The control samples provided by the manufacturer were within the recommended target value and the inter-assay CV based on a pooled serum was 4.4% (*n* = 22).

### 2.4. Statistical Analyses

Baseline descriptive characteristics have been presented as means ± SDs for continuous variables and as counts and percentages for categorical variables. Student’s *t*-tests, Fisher’s Exact tests, and Chi-squared tests were used to examine differences between groups. Pearson’s correlation coefficients were used to assess associations between continuous variables. For all regression analyses, 25OHD values were log transformed (natural logs), these were back transformed to geometric means for the purposes of Figure 3. Linear models were used to analyse the relationships between cord 25OHD and season; maternal 25OHD and PTH; and maternal 25OHD and BMI, maternal skin colour, parity, season of 25OHD measurement, pregnancy (or not), and vitamin D supplement intake. Next, for maternal 25OHD, factors that were significantly associated or considered biologically plausible were then included in a multivariable mixed model analysis. Mixed models included a random intercept for each mother, using BMI (>30 kg/m^2^), vitamin D supplement intake, parity, season of 25OHD measurement, pregnancy, and maternal skin colour as predictors. To examine the predictive potential of very early maternal pregnancy 25OHD status on final maternal 25OHD status at delivery, further modelling was undertaken to graphically illustrate longitudinal variation in maternal 25OHD status based on season of conception. Modelled means of maternal 25OHD status grouped by season of conception, spanning pregnancy (earliest sampling points were at 6 weeks gestation) out to 20 weeks post-partum, centred by date of delivery (gestational ages adjusted relative to date of delivery) were plotted adjusting for all factors included in the mixed model analysis above, excluding only month of measurement, and pregnancy (both variables changing along the *x*-axis). For simplicity, whenever described, and for all analyses undertaken, season has been defined by calendar month: summer: December, January, February; autumn: March, April, May; winter: June, July, August; and spring: September, October, November. 

A 2-sided *p* value of <0.05 was considered significant. All statistical analyses were performed using R version 3.4 [31], using the lme4, and lmerTest libraries [32].

This study was approved by the New Zealand Lower South Regional Ethics Committee (LRS/11/02/007), and the study registered prior to commencement with the Australian New Zealand Clinical Trials Registry at www.anzctr.org.au as ACTRN12611000108910.

## 3. Results.

Baseline characteristics of the 126 women and their infants are presented in Table 1.

There were no differences in basic demographics between those who provided samples at all time points vs. those with missing data points, other than there were fewer births in spring/summer in the “all time points” group 34% vs. 53% respectively, *p* = 0.02. The majority of mothers and their infants had no vitamin D supplement exposure during the study (Table 1).

The longitudinal serum 25OHD and PTH values spanning the three time-points in pregnancy, and two time-points postpartum (out to 20 weeks) in both mothers and infants are shown in Table 2. Table 2 also shows the proportion of mothers at each time point with vitamin D deficiency (25OHD < 50 nmol/L) by season. Maternal vitamin D deficiency was common. In those where both pregnancy and postnatal values were available, deficiency was found at one or more time-points during the full longitudinal study (antenatal plus postnatal) in 65% (52/80), and during pregnancy in 48% (38/80). Infant vitamin D deficiency was also very common, seen in 76% at one or more time-points, with 68% deficient at birth, and a mean cord blood of 41 nmol/L. Mean cord 25OHD was 25 nmol/L greater when season of delivery was in summer (mean ± SD, 57 ± 23 nmol/L), compared to winter (32 nmol ± 16) (*p* < 0.001). At 20 weeks postpartum, the majority remained exclusively breastfed (71% received only breastmilk. No other liquid, solids, or supplements were given. No infants received vitamin D supplements), but increased their 25OHD status to a mean of 57 ± 42 nmol/L. However, 51% remained deficient, and in three infants, there was potentially clinically important secondary hyperparathyroidism, serum PTH values between 12.6 and 29.5 pmol/L (upper limit normal 7 pmol/L). Although normal values for serum PTH in infants have not been consistently defined, these PTH values were accompanied by severe vitamin D deficiency (25OHD concentrations of 4.7, 6.4, and 8.6 nmol/L) and in two infants, elevated alkaline phosphatase levels (352 and 507U/L), all factors that suggest possible vitamin D deficiency rickets. These three infants (only one with dark skin) were all born between late summer and mid-autumn, meaning this 20-week sample was taken in mid-winter/early spring—at the anticipated nadir of 25OHD status.

**Figure 2:**
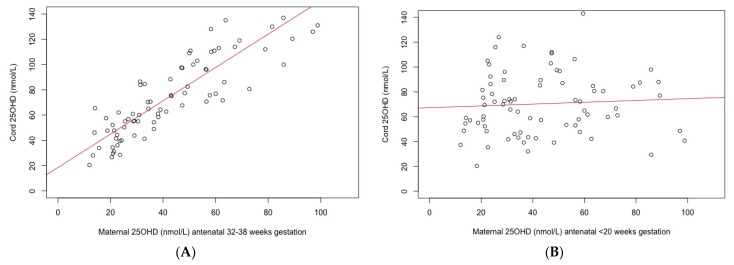
(**A**) Correlation between infant cord 25-hydroxy vitamin D (25OHD) and maternal late pregnancy serum 25OHD (week 32−38), *r*^2^ = 0.87 (95% CI 0.8–0.91); (**B**) Correlation between infant cord 25OHD and maternal early pregnancy (<20-week gestation) serum 25OHD, *r*^2^= 0.06 (95% CI −0.16–0.28).

Positive correlations were observed between infant cord blood 25OHD and two of three maternal 25OHD antenatal sampling time points, as well as at 4 weeks postpartum. The maternal AN3 antenatal time point (32–38 Weeks) was most closely correlated with infant cord blood status, *r*^2^ = 0.87 (95% CI 0.8–0.91), followed by week 4 postpartum, *r*^2^ = 0.81 (95% CI 0.74–0.86), and AN2 (week 20–31), *r*^2^ = 0.4 (95% CI 0.19–0.57). There was no correlation seen between with the maternal AN1 (<20-week gestation) 25OHD and infant cord 25OHD, *r*^2^ = 0.06 (95% CI −0.16–0.28). This is demonstrated in Figure 2. Additionally, there was a weak, negative correlation between maternal 25OHD and PTH (*r*^2^ ranging between −0.12 and −0.31), with little difference in the correlations between the five available maternal time points (both throughout pregnancy and postpartum), with similar overlapping confidence intervals, (spanning −0.52–0.11). The strongest negative correlation between 25OHD and PTH was seen in the infant at 5 months of age, *r*^2^ = −0.35 (95% CI −0.50–−0.16, *p* = 0.0003). No correlation was seen between cord 25OHD and cord PTH, *r*^2^ = −0.11 (95% CI −0.29–0.08, *p* = 0.2).

**Figure 3:**
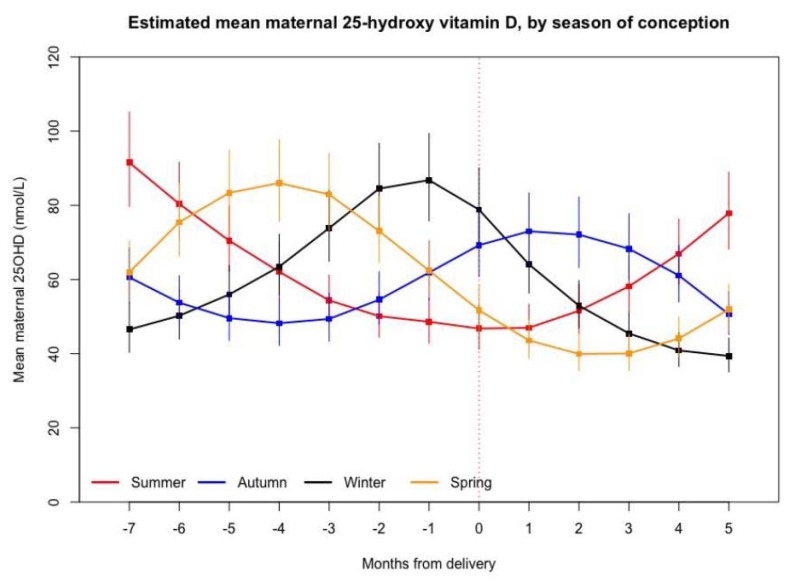
Variation in estimated mean maternal 25-hydroxyvitamin D, by season of conception. Geometric Means ±95% CI presented. Time 0 on the *x*-axis represents time of birth, with negative values the antenatal months pre-delivery (no data was available earlier than 6 weeks gestation), and positive values postnatal months. Season of conception deduced from gestational age. Model adjusting for all variables in Table 3, excluding pregnancy status and season (both variables changing along the *x*-axis). 25OHD, 25-hydroxyvitamin D.

Variables affecting maternal serum 25OHD fully adjusted for season, pregnancy, supplement intake, skin colour, parity, and BMI are shown in Table 3. Due to the significant impact of time of year on 25OHD status, maternal 25OHD status measured over time (gestation and postnatal) by season of conception is shown in Figure 3 demonstrating the variability in serum 25OHD at this latitude over time, and gestation (model adjusted for all variables in Table 3, excluding month and pregnancy stage).

## 4. Discussion

The results of the present study contribute to the growing literature on vitamin D and PTH status during pregnancy and lactation in both mother and offspring, and to the best of our knowledge, are the first longitudinal data from the southern hemisphere. The main findings showed that during pregnancy and out to 5 months postnatally, at one or more time-points, both infant and maternal vitamin D deficiency were very common, especially during winter and spring. Infant cord 25OHD status was most strongly correlated with maternal status towards the end of the third trimester, and importantly had no correlation with maternal status prior to 20 weeks gestation. In addition, pregnancy itself was an independent predictor of maternal vitamin D status with levels being higher at all measured time points throughout pregnancy compared with post-pregnancy values, a finding that remained after adjustment for all other measured variables.

Maternal and infant vitamin D deficiency found in the present study was greater than other comparative longitudinal pregnancy cohorts (rates of 19–33% (25OHD < 50 nmol/L)), albeit these studies were conducted in Scandinavia where dietary fortification and supplementation is more prevalent [19,22]. Our reported rates of deficiency are also greater than the 42% (maternal), and 57% (infant) deficiency rates (25OHD < 50 nmol/L) found in more northern New Zealand cohorts at 36–43° S [18,28]. In fact, deficiency rates in the present study (of generally “low risk” women) are more comparable to the rate reported in a “high risk” multi-ethnic population from Wellington, at 41° S [27]. The significance of the levels of deficiency seen is emphasised by the three otherwise healthy breastfed infants (only one dark skinned) at 20 weeks of age (sampled during mid-winter/spring) who had biochemical values consistent with vitamin D deficiency rickets, a clinical consequence not seen in any participants of the aforementioned studies.

Our findings have important implications for those living in the south of the southern hemisphere. Firstly, the lack of correlation between cord 25OHD and early pregnancy maternal serum 25OHD (Figure 2), raises questions about the utility of screening for maternal vitamin D deficiency in early pregnancy as recommend by regional guidelines [33]. This, combined with the high rates of deficiency found (irrespective of season) in largely “low risk” light skinned European women and infants, means that consideration should be given to strengthening current guidelines, including further thought given to recommending a universal supplementation strategy during pregnancy and exclusive lactation. Currently, New Zealand’s supplementation policy recommends that supplementation be only considered for those who are: dark skinned; completely avoid sun exposure; have liver or kidney disease; or live in southern regions during winter [29]. These data show that without supplementation, these individuals are clearly not achieving optimal vitamin D status throughout pregnancy [29,34]. Furthermore, as pregnancy and lactation span multiple seasons, targeting only pregnant women in the “winter” is also not efficacious. Figure 3, which examines 25OHD status by season of conception, clearly demonstrates this, with first trimester summer participants having the lowest 25OHD status by the time of delivery (Figure 3).

Our longitudinal data highlights that 25OHD status during pregnancy and lactation is influenced by many variables. These include a dominant influence of season, which is well described in the literature in both pregnant and non-pregnant life stages [18,34,35,36]. In addition, maternal 25OHD status appears higher in pregnancy than after delivery, an association that appears to exist beyond that explained by the other variables in our model. It is possible that this association is partially explained by other variables not in the model, such as sun exposure behaviour following pregnancy, but also suggest some of this longitudinal variability may be directly related to the physiological state of pregnancy itself. This would be consistent with other longitudinal pregnancy studies, which have shown a decrease in 25OHD status by the time of delivery [21,24]. Milman et al. found an increase in 25OHD status up to 32 weeks followed by a decrease until delivery [22]. Likewise, Lundqqvist et al. found 25OHD status increased until at least 35 weeks gestation, followed by a fall found at 12 weeks postnatal [19]. Physiological changes in pregnancy may account for this including variation in the vitamin D binding protein (VDBP) which has been shown to increase in pregnancy [24,25,26,27,28,29,30,31,32,33,34,35,36,37]. The possible decline at the end of the third trimester could also reflect increasing 1,25-dihydroxyvitamin D production which is seen at this time [38,39]. Analytical measurement issues may have also contributed to inconsistent results, for instance the detection or not of the C3-epimer of vitamin D [40], which has also been shown to increase in pregnancy [37]. Finally, PTH in pregnancy (and at birth and throughout lactation) in our data was shown to have a relatively weak inverse correlation with 25OHD status. This has also been reported previously [41], and therefore does not appear to be a reliable predictor of adequate 25OHD status during pregnancy.

Sampling of both 25OHD and PTH in a setting of minimal dietary fortification and very little vitamin D supplementation to both mothers and infants throughout pregnancy and postpartum are particular strengths of the present study. Additionally, the longitudinal nature of the data collection, spanning multiple stages of pregnancy to 20 weeks of lactation is another strength, as is the traditionally “low risk” largely ethnically homogeneous population. The latter point, however, is also a limitation, as it remains uncertain how generalizable the findings of this convenience population sample are to other latitudes (particularly those closer to the equator, where fluctuation in 25OHD status with season will be less) and to other ethnic groups. A final limitation is the relatively small sample size, which may have influenced some of our findings.

In conclusion, vitamin D deficiency in women and their infants is very common during pregnancy and lactation in New Zealand at 45° S. The main predictors of maternal vitamin D status were season, supplement use, skin colour, and pregnancy itself. However, the frequency, and at times severity of the vitamin D deficiency seen in this longitudinal cohort raise significant questions regarding the relevance and efficacy of current public health policies for those living in southern New Zealand, particularly the validity of first trimester screening and targeted supplementation for those currently defined “at risk”.

## Figures and Tables

**Table 1:** Baseline characteristics of participants (mother and infant) ^1^.

Characteristics (Total *n* = 126)	
Maternal	
Age, year	32.8 ± 5.0
Ethnicity	
European	104 (83%)
Maori	8 (6%)
Pacific	1 (1%)
Other	13 (10%)
Skin color ^2^	
Very light	67 (53%)
Light	51 (41%)
Intermediate	5 (4%)
Dark	3 (2%)
Primiparous	37 (29%)
Tertiary education ^3^	90 (71%)
Pre-pregnancy BMI, kg/m^2^ Primiparous	25.0 ± 6.2
Smoker	9 (7%)
Antenatal vitamin D supplement use ^4^	35 (28%)
Supplement intake IU/day ^4^	
Consumers only	339 ± 269
Infant	
Gestation, week	39.3 ± 1.1
Gender, male	61 (48%)
Birth weight, g	3506 ± 495
Birth Season	
Spring	30 (24%)
Summer	28 (22%)
Autumn	28 (22%)
Winter	40 (32%)
Skin color ^2^	
Very light	2 (2%)
Light	64 (50%)
Intermediate	51 (40%)
Dark	9 (7%)
Exclusive breast feeding ^5^	90 (71%)
Breastfeeding at week 20	110 (87%)
Infant vitamin D intake, ^6^ IU/day	
Consumers only	96 ± 146

Total (*n* = 126); participants with data at all three antenatal time points (*n* = 80); participants with data at postnatal week 20 (*n* = 66, due to exclusion of those in intervention arms).^1^ Primiparous Values are means ± SDs or *n* (%). ^2^ Underarm “natural” skin colour as determined by skin reflectance using spectrophotometer. ^3^ Completed university or other higher education qualification. ^4^ Maternal supplement use during pregnancy. ^5^ Infant received only breastmilk. No other liquid, solids, or supplements were given over 20 weeks. ^6^ Birth to Week 20 postnatal. Number of infants consuming any formula: *n* = 36.

**Table 2:** Serum total 25-hydroxyvitamin D and parathyroid hormone concentrations in mothers and their infants during pregnancy and lactation (first 20 post-natal weeks) ^1^.

Sampling Occasion	Maternal AN1 (*n* = 80)	Maternal AN2 (*n* = 80)	Maternal AN3 (*n* = 80)	Maternal PN1 (*n* = 123)	Maternal PN2 (*n* = 66)	Infant Cord (*n* = 122)	Infant PN2 (*n* = 66)
Gestation/postnatal week	Week 6–19	Week 20–31	Week 32–38	Week 4	Week 20	Date of birth	Week 20
Serum 25OHD, nmol/L	70 ± 25	78 ± 32	76 ± 34	55 ± 24	59 ± 25	41 ± 21	57 ± 42
Serum PTH, pmol/L	1.7 ± 0.7	1.6 ± 0.8	1.5 ± 0.7	3.5 ± 1.7	3.8 ± 1.7	0.5 ± 0.5	2.8 ± 3.9
Proportion with 25OHD deficiency (<50 nmol/L) by season, %							
Spring	26% (8/31)	56% (9/16)	50% (6/12)	59% (19/32)	50% (9/18)	93% (27/29)	39% (7/18)
Summer	5% (1/19)	6% (1/17)	6% (1/16)	20% (4/20)	9% (1/11)	37% (10/27)	0% (0/11)
Autumn	21% (3/14)	7% (2/30)	18% (5/28)	41% (12/29)	36% (5/14)	50% (14/28)	14% (2/14)
Winter	50% (8/16)	47% (8/17)	33% (8/24)	57% (24/42)	70% (16/23)	84% (32/38)	87% (20/23)

^1^ Primiparous Values are means ± SDs or % (*n*); AN, Antenatal; PN, Postnatal; 25(OH)D, 25-hydroxyvitamin D; PTH, parathyroid hormone.

**Table 3:** Predictors of maternal serum 25-hydroxyvitamin D status during pregnancy and lactation (first 20 post-natal weeks) ^1^.

Predictor	Estimate	95% Confidence Interval (CI)	*p*-Value
Season			
Summer	Ref.		
Autumn	0.72	0.67–0.78	<0.001
Winter	0.58	0.54–0.63	<0.001
Spring	0.7	0.65–0.76	<0.001
Pregnancy ^2^	1.25	1.17–1.33	<0.001
Supplement Use	1.2	1.09–1.33	<0.001
Skin Colour ^3^			
Very Light	Ref.		
Light	1.07	0.95–1.21	0.29
Intermediate	1.01	0.74–1.36	0.97
Dark	0.55	0.38–0.80	0.003
First Pregnancy	0.88	0.78–1.01	0.07
BMI ^4^ > 30 kg/m^2^	0.95	0.85–1.05	0.32

^1^ Multivariable mixed model analysis; ^2^ Pregnancy life stage compared to postnatal (birth to 20 weeks); ^3^ Skin colour as determined by spectrophotometry; ^4^ BMI, Body mass index (13% > 30 kg/m^2^).
